# The role of upper airway ultrasonography in tracheal necrosis diagnosis: a case report

**DOI:** 10.1186/s13089-024-00385-2

**Published:** 2024-07-12

**Authors:** Mas Fazlin Mohd Jailaini, Mohd Jazman Che Rahim, Wan Aireene Wan Ahmed, Shaik Farid Abdull Wahab, Mohamed Faisal Abdul Hamid, Fahrin Zara Mohammad Nasseri

**Affiliations:** 1 Faculty of Medicine, Universiti Kebangsaan Malaysia, Kuala Lumpur, Malaysia; 2 School of Medical Sciences, Universiti Sains Malaysia Health Campus, Kota Bharu, Kelantan Malaysia; 3 Hospital Universiti Sains Malaysia, Kota Bharu, Kelantan Malaysia

**Keywords:** Tracheal necrosis, Airway ultrasound, Upper airway obstruction, Airway management

## Abstract

**Background:**

Tracheal necrosis post endotracheal intubation is a rare life-threatening disease that can compromise airway patency. We demonstrated a novel usage of upper airway ultrasonography (USG) to diagnose tracheal necrosis.

**Case presentation:**

A middle-aged smoking male presented with productive cough, noisy breathing and exertional dyspnea for 2 weeks. He was intubated one month prior due to a traumatic brain injury. Upper airway USG findings showed irregular air-mucosal interface (AMI) and comet tail artefacts over the 1st and 2nd tracheal ring. A direct laryngoscopy in the operating room showed thick mucopus inferior to the vocal cords, with necrotic tracheal cartilages and debris obstructing the airway. He was successfully treated with parenteral antibiotics, wound debridement and tracheostomy.

**Conclusion:**

Our case highlights the first documented USG findings of tracheal necrosis. Upper airway USG serves as a potential diagnostic modality in managing the condition.

**Supplementary Information:**

The online version contains supplementary material available at 10.1186/s13089-024-00385-2.

## Background

Tracheal necrosis post endotracheal intubation is a rare but well-documented condition. Published data is mostly from case reports with 182 reported cases from 1966 to 2007 [1]. Factors that may lead to tracheal necrosis include prolonged intubation, elevated cuff pressure, tracheal blood supply disruption [2], bacterial and fungal infection [3], immunocompromised state, diabetes mellitus, upper airway surgery, esophageal surgery [4] and thyroidectomy [5]. Patients present with dysphonia, cough, dyspnea and respiratory failure. Histopathology findings include severe tracheal inflammation, pseudomembrane formation [3], mucosal ulceration and ultimately tracheal cartilage destruction [5]. Tracheal necrosis may lead to upper airway obstruction and tracheal perforation. Its treatment involves securing the airway with tracheostomy, debridement of necrotic tissue, acute infection treatment, management of co-morbidities and airway reconstruction once the infection has resolved [3, 4]. An early low-dose systemic corticosteroid treatment should be considered to reduce the risk of tracheal stenosis needing airway resections [6]. Tracheal necrosis poses a major challenge in airway management whereby ensuring a patent airway is the foremost step in any resuscitation attempts. We report the ultrasonographic (USG) upper airway findings of a patient with a tracheal necrosis post endotracheal intubation.

### Case presentation

A middle-aged active smoker presented with productive cough with greenish sputum, reduced effort tolerance and noisy breathing for 2 weeks. He had no fever, throat discomfort or dysphagia.

One month prior, he was intubated for 4 days after a traumatic brain injury following a motor vehicle accident. He was extubated and discharged well after 2 weeks of admission. He was also treated for *Streptococcus mitis* bacteremia with 2 weeks of IV amoxycillin clavulanate and benzyl penicillin.

He had hypertension and type 2 diabetes mellitus (DM) which were not under any treatment as the patient opted for dietary control and observation. His hemoglobin A1C (HbA1C) upon admission was 10.5%.

On examination, he was conscious, alert, mildly tachypnoeic at rest with respiratory rate of 24 breaths per minute and saturating between 90 and 95% on room air. There was an audible stridor. Vital signs were otherwise normal. There was no recorded fever. Capillary blood sugar was 9.0 mmol/L. The rest of the physical examination was unremarkable. There were no evident micro- or macrovascular complications of DM.

Blood investigation showed a normal total white cell count (7.4 × 10^9^/L). C-reactive protein (CRP) was elevated (6 mg/dL). His renal and liver profile were normal. Arterial blood gas showed no hypoxia or acidosis.

**Fig. 1:**
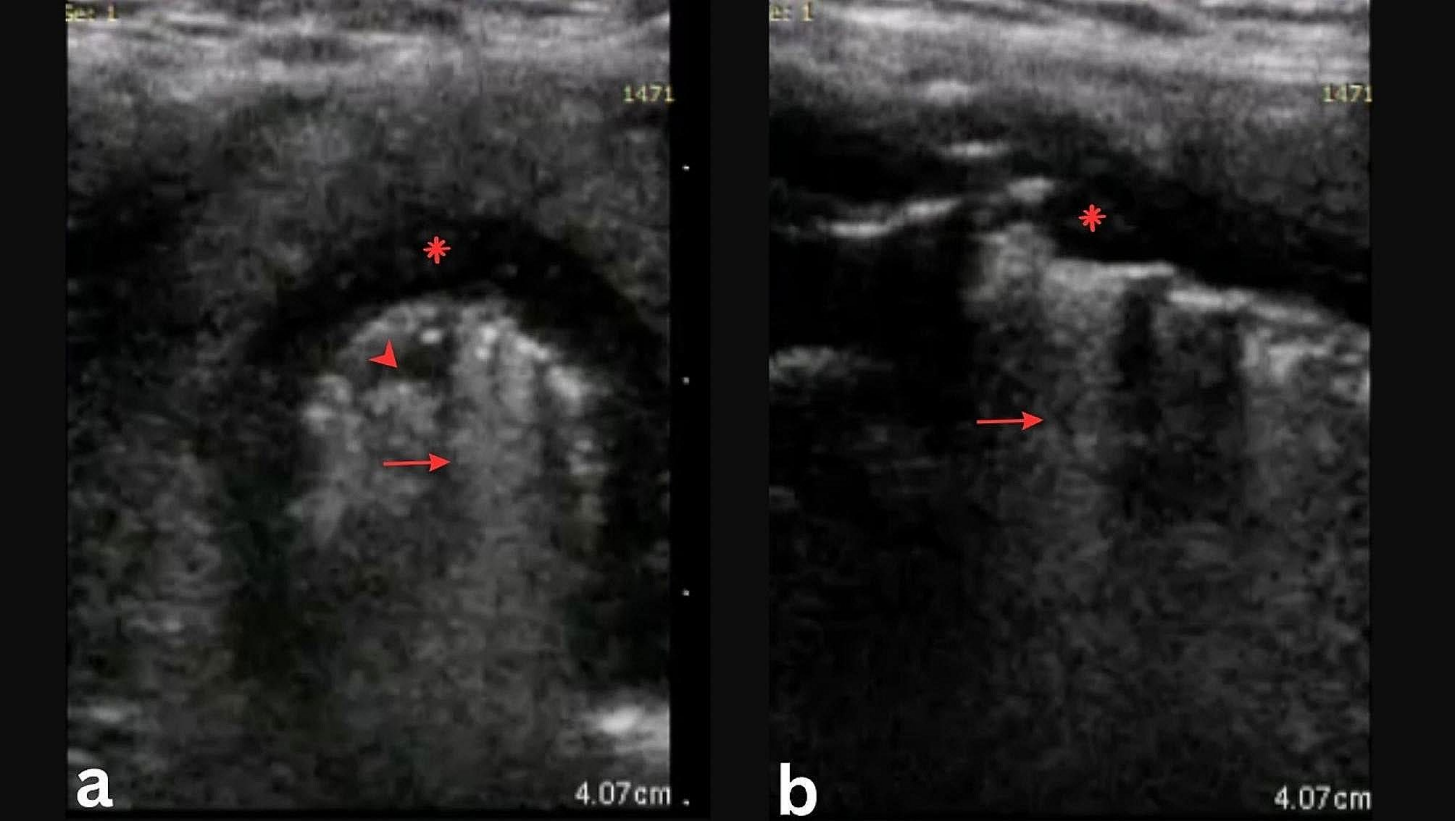
(**a**) Axial USG view of T1. Note the jagged appearance (arrowhead) of the AMI. (**b**) Sagittal view of the trachea. Note the comet tail artefact (arrow) beneath T1 (asterix)

A flexible laryngoscopy showed thick mucopus inferior to the vocal cords which was difficult to remove with suction. There were parts of necrotic tracheal cartilage embedded in mucopus and debris obstructing the airway. A tracheal long segment upper airway obstruction with impending airway emergency was suspected. A bedside upper airway USG using the handheld Vscan Extend machine (GE Healthcare, Chicago, IL 60,661, USA) showed an irregular, jagged air-mucosal interface (AMI) at the level of the 1st (T1) and 2nd (T2) tracheal ring (Fig. 1). Comet tail artefacts were also seen beneath the abnormal AMI. Flexible bronchoscopy using a large-bore scope was not attempted due to fear of tracheal collapse.

**Fig. 2:**
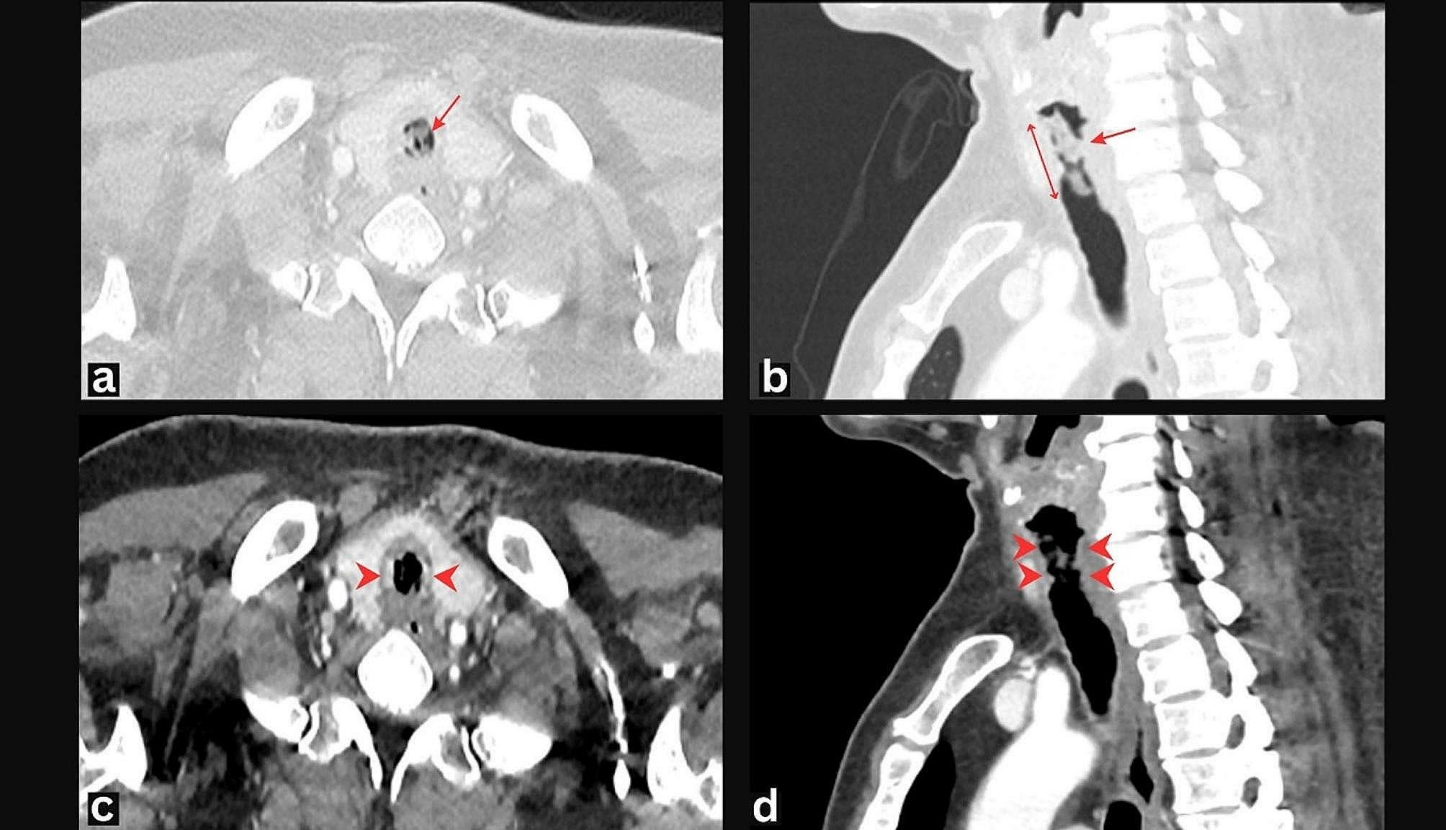
(**a**-**d**) Contrast enhanced CT of the neck and thorax in axial and sagittal view at thyroid level. Lung window (**a** and **b**) demonstrates a focal, short-segment endoluminal mucous secretion within the trachea (arrow) at the subglottic region, along a length of 3.5 cm (double arrow). The tracheal wall is irregular with circumferential thickening (arrowhead) and luminal narrowing on soft tissue window (c and d)

Computed tomography (CT) of the neck and thorax was showed a focal, short-segment endoluminal mucous secretion within the trachea at the subglottic region at the level of the 6th and 7th cervical vertebrae, 8 cm above the carina, along a length of 3.5 cm (Fig. 2). Tracheal wall irregularity was seen in this region with minimal wall thickening. There was also a poor fat plane. No goiter or lymphadenopathy were found. The lungs and mediastinum were normal.

**Fig. 3:**
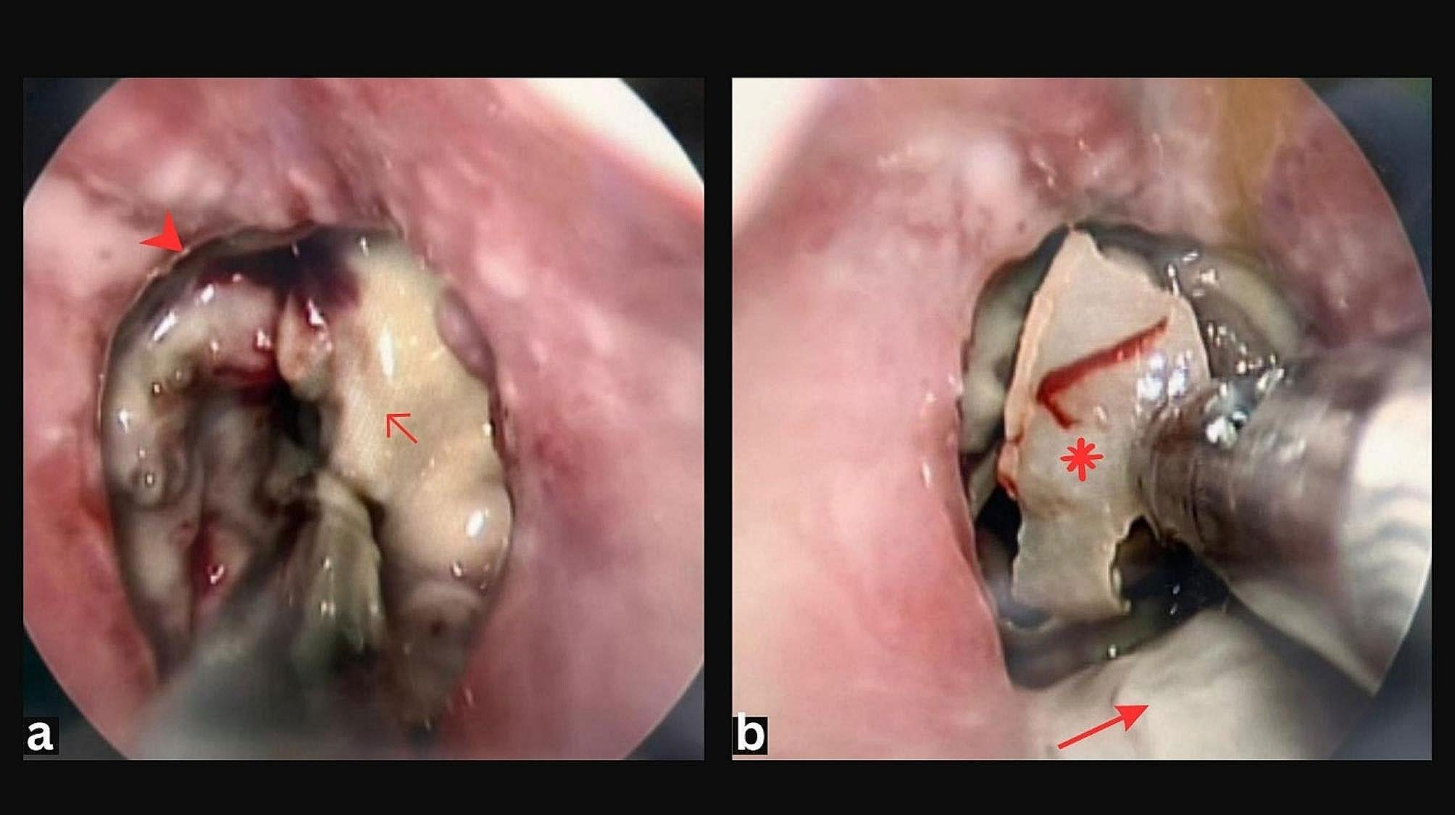
(**a**-**b**) Direct laryngoscopy. Note the irregular tracheal mucosa (arrowhead) with pus dan debris adhering to the tracheal wall (short arrow). Necrotic tracheal cartilage (asterix) is seen in the trachea, causing obstruction. The posterior tracheal wall is covered with slough (long arrow)

A direct laryngoscopy under general anaesthesia (GA) was done. Removal of slough and necrotic tissue with tracheostomy was done to secure the airway (Fig. 3). Intraoperatively, a necrotic segment of the trachea was seen 2 cm from the free edge of the vocal cord, involving the 1st and 2nd tracheal cartilage. The inferior edge of the necrotic part was 5.5 cm from the carina. The bronchoscopy finding after the surgery was unremarkable.

Histopathology samples showed a fragment of benign hyaline cartilage displaying areas of dystrophic calcification. There was mild superficial infiltration by neutrophils admixed with fibrin. Parts of the superficial cartilage appeared pale and unhealthy with loss of chondrocytes within the lacunae. There was no granuloma and no evidence of malignancy. Cultures for fungus, mycobacteria and bacteria showed no growth.

The patient was discharged 1 week after the surgery. He had completed a 1-week course of amoxycillin clavulanate and nebulised ciprofloxacin.

His condition was complicated with tracheal stenosis, requiring multiple surgical procedures. Subsequent intraoperative cultures from the tracheal tissue grew *Streptococcus mitis* and *Pseudomonas aeruginosa*, both were sensitive strains and adequately treated with suitable antibiotics.

## Discussion

Tracheal pathologies are routinely assessed using neck and chest X-ray and computed tomography (CT) of the neck and thorax. The former view can be obscured by the overlying mediastinum and bony structures. CT allows detailed and unhindered view of tracheobronchial lesions [7]. However, it is not readily available in rural healthcare settings. Bronchoscopy allows direct visualisation of the upper airway, determination of the lesion extent and various procedures to be carried out such as biopsy, stenting and irradiation. Nevertheless, performing bronchoscopy requires properly-trained personnel in suitable settings on suitable patients in order to prevent complications such as bronchospasm, hypoxemia, oversedation and bleeding [8]. Thus, a more accessible, practical and rapid diagnostic modality is needed.

Point-of-care USG (POCUS) of the upper airway offers an opportunity to diagnose tracheal necrosis faster, allowing early appropriate airway management. Upper airway USG has been used in assessing soft tissue pathologies before. These include measuring air column width differences (ACWD) in intubated patients with suspected laryngeal edema [9], assessing tracheal wall thickness in smoke inhalation patients [10], detecting an upper airway mass [11] and detecting features of laryngeal trauma [12]. Abnormal upper airway signs include ACWD > 1.6 mm in intubated patients [9], the disruption of AMI or cartilage continuity [12], the presence of hematoma or soft tissue edema and tracheal wall thickness > 1.2 mm (female) or > 1.5 mm (male) [10]. Irregular AMI and comet tail artefacts have never been documented before in upper airway USG.

AMI in the trachea appears as a straight, hyperechoic line immediately beneath the hypoechoic upper airway cartilages, as a result of a greatly different attenuation coefficients of air and the cartilage which produces a strong echo [13]. Structures beneath the AMI are not seen due to the acoustic shadowing. The same concept applies to the pleural line in lung USG. The pleural line appears as a hyperechoic line due to the different attenuation coefficients of the thoracic wall’s soft tissue and the air beneath the visceral pleura.

In lung consolidation, the pleural line appears jagged due to the air in the alveoli being replaced with fluid (exudates, pus or blood) [14]. The increase in the interstitial fluid results in B lines formation. In our case, the jagged appearance of the AMI was likely due to the irregular inner surface of the necrotic tracheal cartilage. The comet tail artefacts were probably formed due to the air molecules coupling with the exudative fluid on the tracheal wall.

Tracheal necrosis is a rare but dangerous condition. The delay in its detection or inadvertent suctioning may cause tracheal collapse and death. Our case highlighted a novel use of USG in assessing tracheal necrosis. Despite having a lower resolution, our handheld USG machine clearly showed the pathologic USG findings. The USG findings correlated well with the CT and intraoperative findings.

Despite its potential as a rapid modality in upper airway disease diagnosis, upper airway USG has its limitations. Performing it requires adequate training and thorough understanding of the sonoanatomy and sonopathology of the upper airway. USG is not able to assess the intrathoracic trachea due to the obstructing sternum and aerated lung parenchyma. Therefore, USG remains as an adjunct diagnostic modality in the management of tracheal necrosis. Further studies are needed to assess its diagnostic accuracy and feasibility in clinical practice.

### Electronic supplementary material

Below is the link to the electronic supplementary material.

Supplementary Material 1

## Data Availability

All materials are included in this submission.

## Abbreviations

USG: Ultrasonography
DM: Diabetes mellitus
HbA1C: Hemoglobin A1C
CRP: C-reactive protein
AMI: Air-mucosal interface
T1: First tracheal cartilage
T2: Second tracheal cartilage
CT: Computed tomography
POCUS: Point-of-care USG
ACWD: Air column width differences
GA: General anaesthesia
